# Plantar cutaneous leiomyosarcoma: an entity of rare presentation and diagnostic challenge^☆^

**DOI:** 10.1016/j.abd.2026.501474

**Published:** 2026-10-30

**Authors:** Edgar Fermín Yan-Quiroz, Thiara Charlene Carranza-Cabanillas, Zaida Urata-Guzmán, Eliani Leydi Silvana Pérez-Moran, Renan Vargas-Morales, José Richard Tenazoa-Villalobos

**Affiliations:** a Virgen de la Puerta High Complexity Hospital, EsSalud, Trujillo, La Libertad, Peru; b Faculty of Medicine, Universidad Privada Antenor Orrego, Trujillo, La Libertad, Peru; c Victor Lazarte Echegaray Hospital, EsSalud, Trujillo, La Libertad, Peru

Dear Editor,

Cutaneous Leiomyosarcoma (CLMS) is a rare malignancy of smooth muscle origin, accounting for 2% to 3% of all cutaneous soft-tissue sarcomas and 0.04% of all malignancies.^1^ It is the third most common cutaneous sarcoma, after dermatofibrosarcoma protuberans and Kaposi's sarcoma.^2^ This case report presents a 32-year-old woman with left plantar cutaneous leiomyosarcoma and examines key clinical, diagnostic, and therapeutic considerations for this rare neoplasm. The patient demonstrated a favorable outcome, with no evidence of recurrence following treatment.

A 32-year-old woman with no significant medical history presented with a nodule on the sole of the left foot. Physical examination identified an elevated, mobile, well-defined, and painless lesion in the internal paramedial middle third of the left plantar region. Ultrasound revealed a heterogeneous nodular lesion measuring 6.5 × 18 × 28 mm, with a positive Doppler signal and no adjacent structure involvement.

Local resection of the left plantar tumor was performed. Intraoperative assessment revealed a firm, well-organized tumor measuring 3 × 2 cm, without defined borders, located in the subcutaneous and dermis planes of the plantar arch. Histopathological analysis identified a malignant mesenchymal neoplasm consistent with sarcoma, histological grade 2, with mitoses in 10 × 400 High-Power Fields (HPF), necrosis, and no evidence of lymphovascular invasion. Immunohistochemistry showed positivity for vimentin, actin, and caldesmon; negativity for pankeratin, desmin, CD34, Melan A, and HMB45; and a Ki-67 index of 15% (Fig. 1). These findings supported a diagnosis of pleomorphic leiomyosarcoma.

**Fig. 1:**
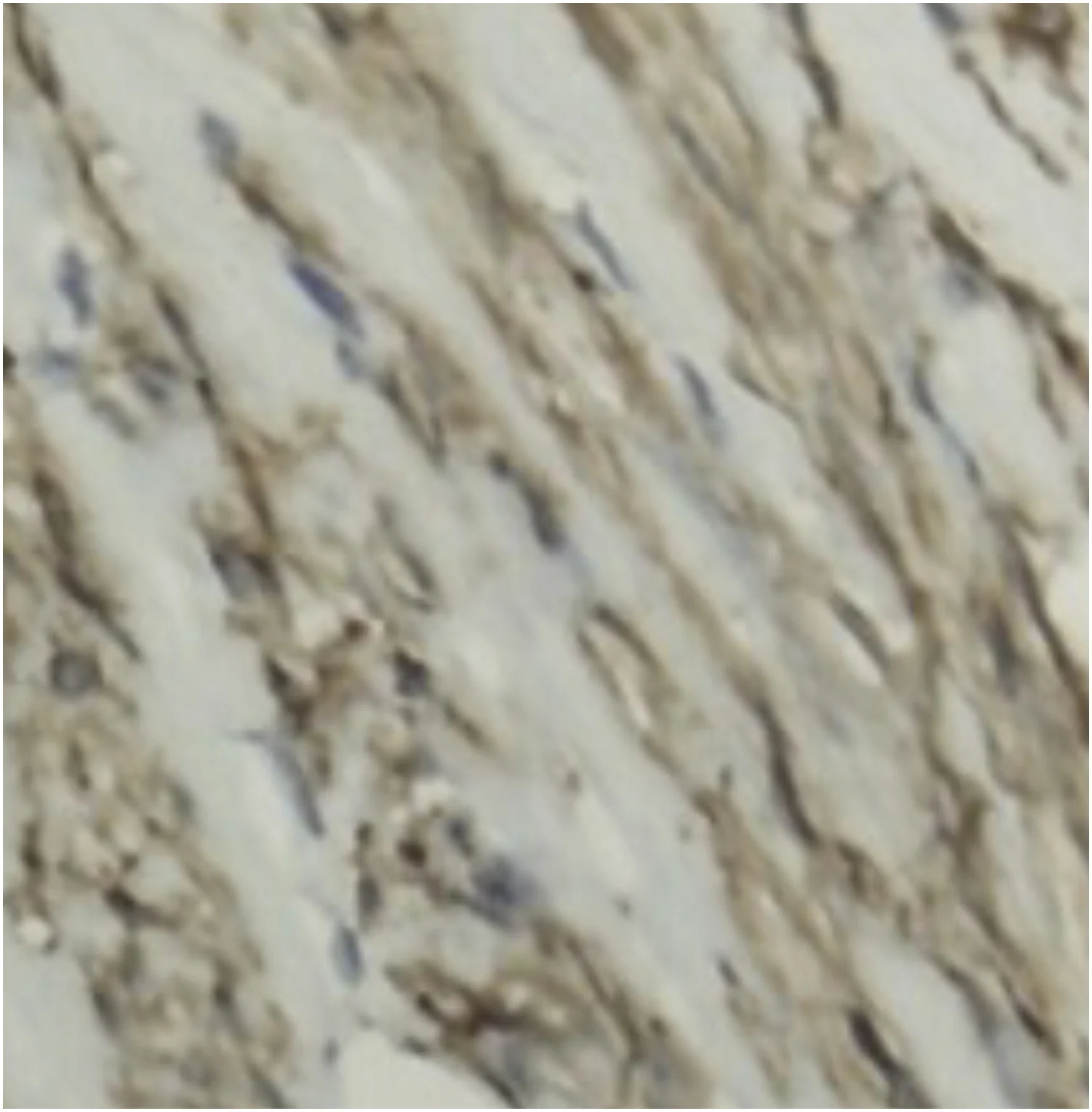
Biopsy immunohistochemistry. Caldesmon (+).

Two months after surgery, a multilobed lesion measuring 3 × 3 cm developed at the operative site. Contrast-enhanced computed tomography of the chest, abdomen, and pelvis showed no evidence of metastasis. Contrast-enhanced magnetic resonance imaging of the left foot identified a lesion in the medial plantar region, measuring 18 × 12 mm, with infiltration of the plantar muscle tendon (Fig. 2). Due to persistent disease, a wide local resection was performed. Microscopic examination revealed a malignant fusocellular mesenchymal neoplasm, consistent with pleomorphic leiomyosarcoma, measuring 2.5 cm and classified as histological grade 3, without evidence of necrosis. There were (Fig. 3) 50 mitoses per 10 × 400 HPF, and no lymphovascular or perineural invasion was observed. Surgical margins measured 0.8 cm from the neoplasm, except for the deep margin, which was in contact with the lesion. Immunohistochemistry demonstrated positivity for vimentin, smooth muscle actin, and CD68; negativity for desmin, caldesmon, CD34, and S100. The Ki-67 index was 20% (Fig. 4).

**Fig. 2:**
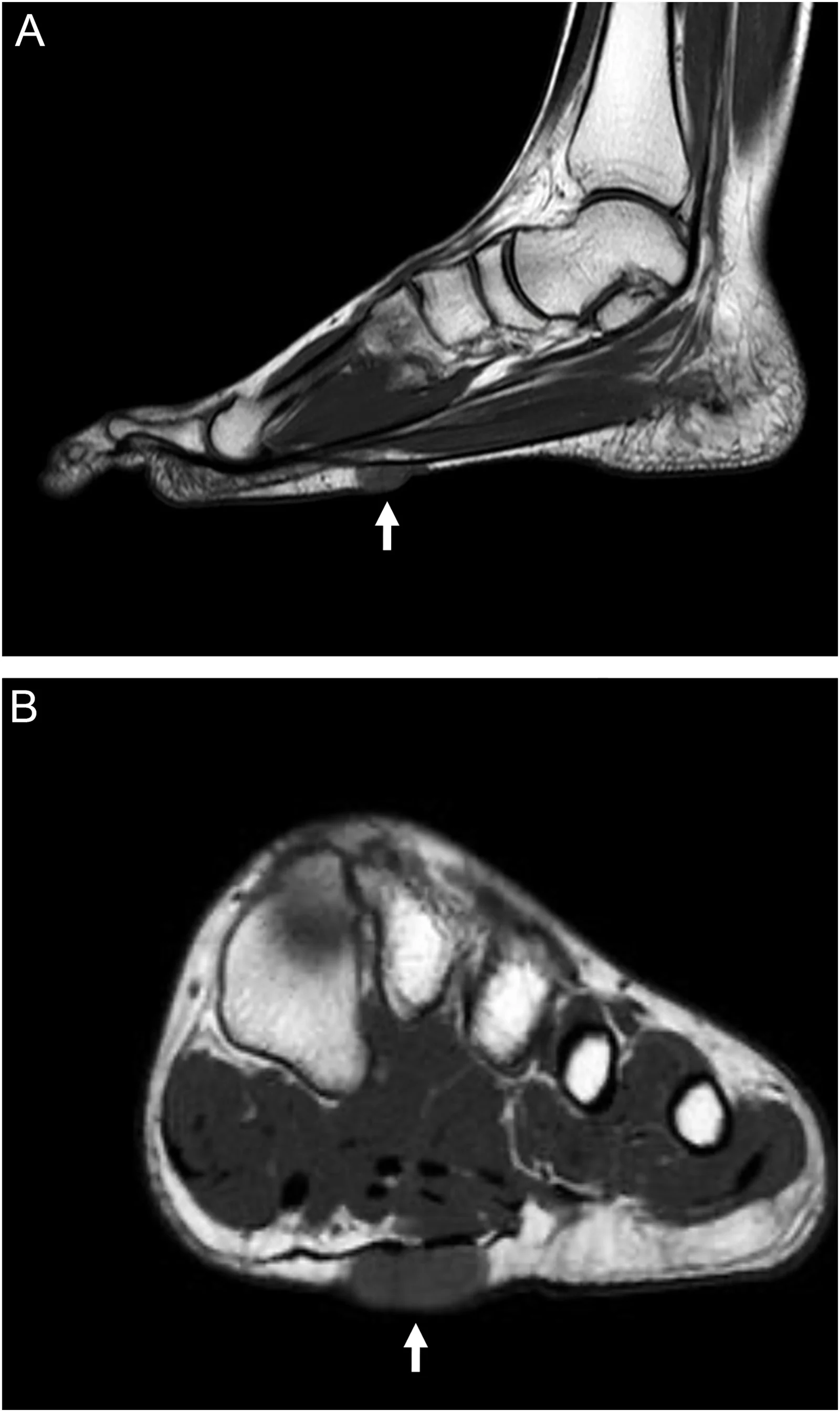
Magnetic resonance imaging of the left foot. (A) Sagittal section. (B) Cross-section. A lobed lesion measuring 18 × 12 mm in the medial plantar region demonstrates contrast enhancement (arrow).

**Fig. 3:**
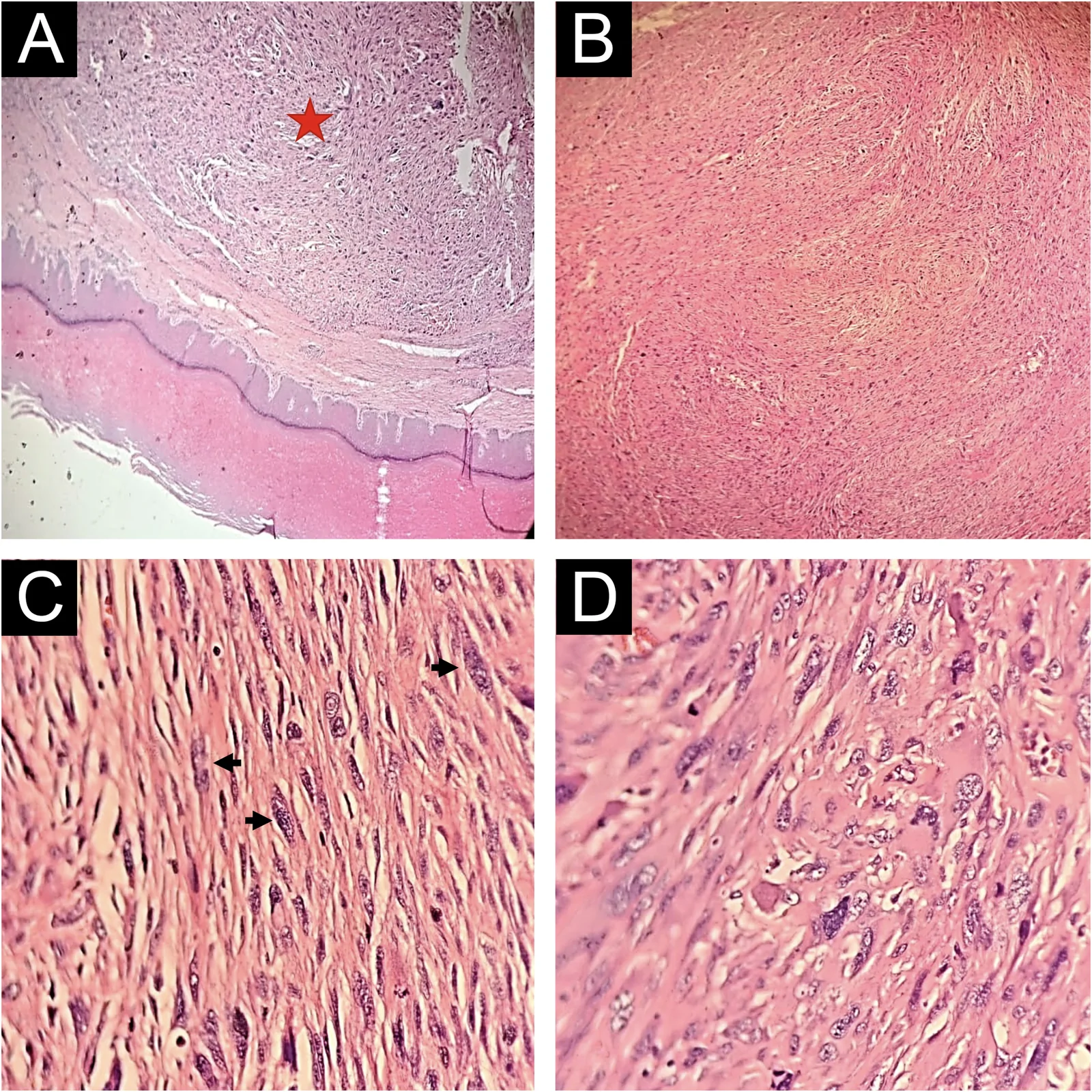
Microscopy, hematoxylin and eosin staining. (A) 4 × . Acral skin from the sole of the foot with a subcutaneous lesion (star). (B) 4 × . Fascicles of eosinophilic spindle cells. (C) 40 × . Fusiform cells with blunt, cigar-shaped nuclei and variable pleomorphism (arrow). (D) 40 × . Spindle cells with eosinophilic cytoplasm.

**Fig. 4:**
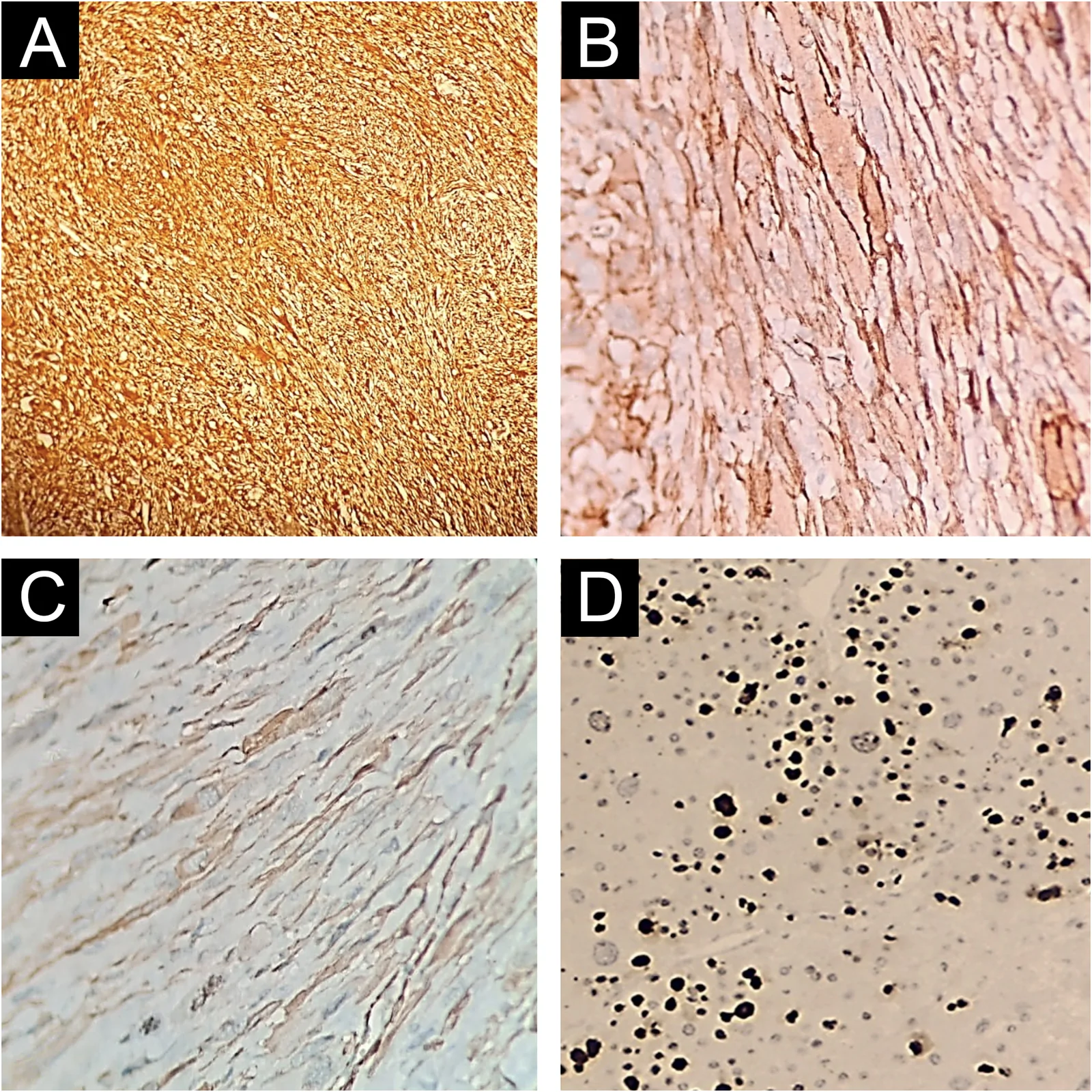
Immunochemistry. (A) Vimentine (+). (B) Smooth muscle actin (+). (C) Smooth muscle actin (+), area of minor staining. (D) Ki-67 (+) 20%.

Postoperative diagnosis confirmed left plantar cutaneous pleomorphic leiomyosarcoma. The postoperative course was favorable, and the patient subsequently received radiotherapy at a total dose of 6000 cGy over 30 sessions. Continued surveillance has revealed no evidence of recurrence or metastasis six months following the initial diagnosis.

The aetiology of Cutaneous Leiomyosarcoma (CLMS) remains unclear, although associations have been reported with trauma, burns, venous stasis, and lupus vulgaris. Plantar localisation may be associated with a higher likelihood of recurrent trauma, which could contribute to tumour development.^3^, ^4^ CLMS may originate from the smooth muscle of blood vessels, cutaneous erector muscles, or through differentiation of mesenchymal cells.^5^, ^6^ It is hypothesized that pluripotent mesenchymal stem cells in fascia, connective tissue, or vascular pericytes acquire DNA mutations, such as p53 loss, and subsequently differentiate aberrantly into smooth muscle lineages.^6^

CLMS is characterized by a proliferation of spindle cells arranged in interlacing fascicles, with eosinophilic cytoplasm and blunt, cigar-shaped nuclei. Immunohistochemistry typically demonstrates positivity for Smooth Muscle Actin (SMA), desmin, and vimentin.^1^ High-risk features include high histological grade (2 or 3), elevated mitotic rate, presence of necrosis, and tumor size greater than 5 cm. These factors are associated with an increased risk of locoregional recurrence (37%) and metastasis (21% to 62%). In contrast, rhabdomyosarcoma is identified by skeletal muscle differentiation and typically expresses myogenin and MyoD1. Malignant fibrohistiocytoma is primarily a diagnosis of exclusion and lacks the specific smooth muscle differentiation, such as caldesmon positivity, seen in cutaneous leiomyosarcoma. Malignant myofibroblastic tumors generally exhibit myofibroblastic rather than pure smooth muscle features; although they are often SMA positive, they usually lack the characteristic cigar-shaped nuclei and the more specific smooth muscle markers observed in cutaneous leiomyosarcoma.^6^, ^7^, ^8^

The differentiation and progression of CLMS are thought to originate from pluripotent mesenchymal stem cells within the fascia or connective tissue. Although CLMS typically expresses desmin, the pleomorphic subtype may lack this marker, as documented in the literature. In the present case study, a recurrent pleomorphic tumor exhibited additional loss of caldesmon expression as it progressed from histological Grade 2 to Grade 3. CLMS is generally classified into two main forms: dermal (purely cutaneous), which is usually less aggressive, and the subcutaneous form, as demonstrated in this case, which involves both the subcutaneous and dermal planes, tends to be larger, and is associated with a higher risk of recurrence and metastasis. Classic CLMS is characterized by spindle cells arranged in interlacing fascicles with blunt, cigar-shaped nuclei. In contrast, pleomorphic CLMS is defined as a malignant fusocellular mesenchymal neoplasm.^9^

Wide local resection is the standard of care for soft-tissue sarcomas of the extremities, with a recommended safety margin of at least 1 cm for cutaneous and subcutaneous leiomyosarcomas to reduce the risk of recurrence. In the foot, where neurovascular structures and bone are in proximity, narrower margins (less than 5 mm) may be acceptable if natural anatomical barriers such as fascia or periosteum are present.^8^, ^10^

Postoperative radiotherapy is recommended when final surgical margins are 1 cm or less, and no intact fascial plane serves as a barrier. Although margin-negative (R0) resection is the goal, narrow margins are associated with local recurrence rates similar to those of positive margins when not supplemented with radiotherapy.^11^

Plantar leiomyosarcoma is a rare malignancy. Wide resection with negative margins remains the primary treatment strategy, and radiotherapy is indicated for high-risk cases. Comprehensive resection with adequate margins is recommended for all soft tissue tumors. Multidisciplinary management is essential for achieving optimal outcomes in the treatment of this neoplasm.

## ORCID ID

Edgar Fermín Yan-Quiroz: 0000-0002-9128-4760

Thiara Charlene Carranza-Cabanillas: 0009-0009-2081-3213

Zaida Urata-Guzmán: 0000-0002-6017-2977

Eliani Leydi Silvana Pérez-Moran: 0009-0009-9060-3377

Renan Vargas-Morales: 0000-0003-3948-4949

## Financial support

None declared.

## Authors’ contributions

Edgar Fermín Yan-Quiroz: Effective participation in research orientation; manuscript critical review; preparation and writing of the manuscript; study conception and planning.

José Richard Tenazoa-Villalobos: Effective participation in research orientation; manuscript critical review; preparation and writing of the manuscript; study conception and planning.

Eliani Leydi Silvana Pérez-Moran: Preparation and writing of the manuscript.

Zaida Urata Guzmán: Preparation and writing of the manuscript.

Thiara Charlene Carranza-Cabanillas: Preparation and writing of the manuscript.

Renan Vargas-Morales: Preparation and writing of the manuscript.

## Research data availability

Does not apply.

## Conflicts of interest

None declared.
